# Supplementary material for: Integrated View of Baseline Protein Expression in Human Tissues
Source: J Proteome Res. 2022 Dec 28;22(3):729–42. doi: 10.1021/acs.jproteome.2c00406 (PMC9990129; doi:10.1021/acs.jproteome.2c00406)
Supplement: Supplementary file 1 — pr2c00406_si_001.pdf [file pr2c00406_si_001.pdf]

TITLE

An integrated view of baseline protein expression in human tissues

AUTHORS

Ananth Prakash[1,2,*], David García-Seisdedos[1], Shengbo Wang[1], Deepti Jaiswal Kundu[1], Andrew Collins[3], Nancy George[1], Pablo Moreno[1], Irene Papatheodorou[1,2], Andrew R. Jones[3,*], Juan Antonio Vizcaíno[1,2 *]

[1] European Molecular Biology Laboratory - European Bioinformatics Institute (EMBL-EBI), Wellcome Genome Campus, Hinxton, Cambridge, CB10 1SD. United Kingdom.

[2] Open Targets, Wellcome Genome Campus, Hinxton, Cambridge, CB10 1SD. United Kingdom.

[3] Institute of Systems, Molecular and Integrative Biology, University of Liverpool, Liverpool L69 7ZB, United Kingdom.

*Corresponding authors.

Dr. Ananth Prakash. European Molecular Biology Laboratory, European Bioinformatics Institute (EMBL-EBI), Wellcome Trust Genome Campus, Hinxton, Cambridge, CB10 1SD, UK. Phone: + 44 (0) 1223 492610. Email: ananth@ebi.ac.uk

Prof. Andrew R. Jones. Institute of Systems, Molecular and Integrative Biology, University of Liverpool, Liverpool L69 7ZB, United Kingdom. Email: jonesar@liverpool.ac.uk

Dr. Juan Antonio Vizcaíno. European Molecular Biology Laboratory, European Bioinformatics Institute (EMBL-EBI), Wellcome Trust Genome Campus, Hinxton, Cambridge, CB10 1SD, UK. Phone: + 44 (0) 1223 492686. Email: juan@ebi.ac.uk

TABLE OF CONTENT

S-2 Table of Content

S-3 Figure S1. Distribution of common reverse protein decoy hits across the number of datasets.

S-4 Figure S2. PCA of FOT normalised iBAQ protein abundances (samples without bin transformation) of (A) brain samples coloured by tissues and (B) and datasets (C) and heart samples coloured by tissues and (D) datasets.

S-5 **Figure S3.** PCA of the heart samples using Combat normalisation where samples are coloured by (A) tissues and (B) datasets. Similarly PCA of heart samples using Limma normalisation where samples are coloured by (C) tissues and (D) datasets.

S-6 **Figure S4**. PCA of brain samples using Combat normalisation where samples are coloured by (A) tissues and (B) datasets Similarly PCA of brain samples using Limma normalisation where samples are coloured by (C) tissues and (D) datasets.

S-7 **Figure S5.** Correlation of protein abundances across various organs compared between ProteomicsDB and this study.

S-8 **Figure S6.** Comparison of protein expression between Jiang et al. (2020) (using TMT labelling method) and data presented in this study (label-free method).

Figure S7. Protein detection comparison between Human Protein Atlas and the results included in this manuscript.

S-9 **Figure S8.** The randomised edit distance difference matrix showing the differences in the edit distances calculated for various organs between this study and the data from Human Protein Atlas.

S-10 **Figure S9.** Proteomics experiments home page in Expression Atlas.

Figure S10. Result page for an example proteomics data.

S-11 **Figure S11.** Display of protein abundances across samples in Expression Atlas.

S-12 **Figure S12.** Expression Atlas 'Downloads' tab.

S-13 Supplementary Tables:

Supplementary Table 1: Protein groups from all datasets that are mapped to more than one Ensembl Gene ID.

Supplementary Table 2: Median protein abundances (in ppb) for each protein group across various tissue samples in each organ.

Supplementary Table 3: Median binned protein abundances across various organs.

Supplementary Table 4: Table showing the comparison of protein and peptide identification numbers across various datasets with the reported ones in their respective original publications.

Supplementary Table 5: 'Organ-enriched' and 'Group-enriched' elevated proteomes in various organs.

Supplementary Table 6: Gene Ontology enrichment analysis of 'organ-enriched' and 'group-enriched' proteins.

Supplementary Table 7: Reactome pathway-enrichment analysis of "organ-enriched" and "group-enriched" proteins.

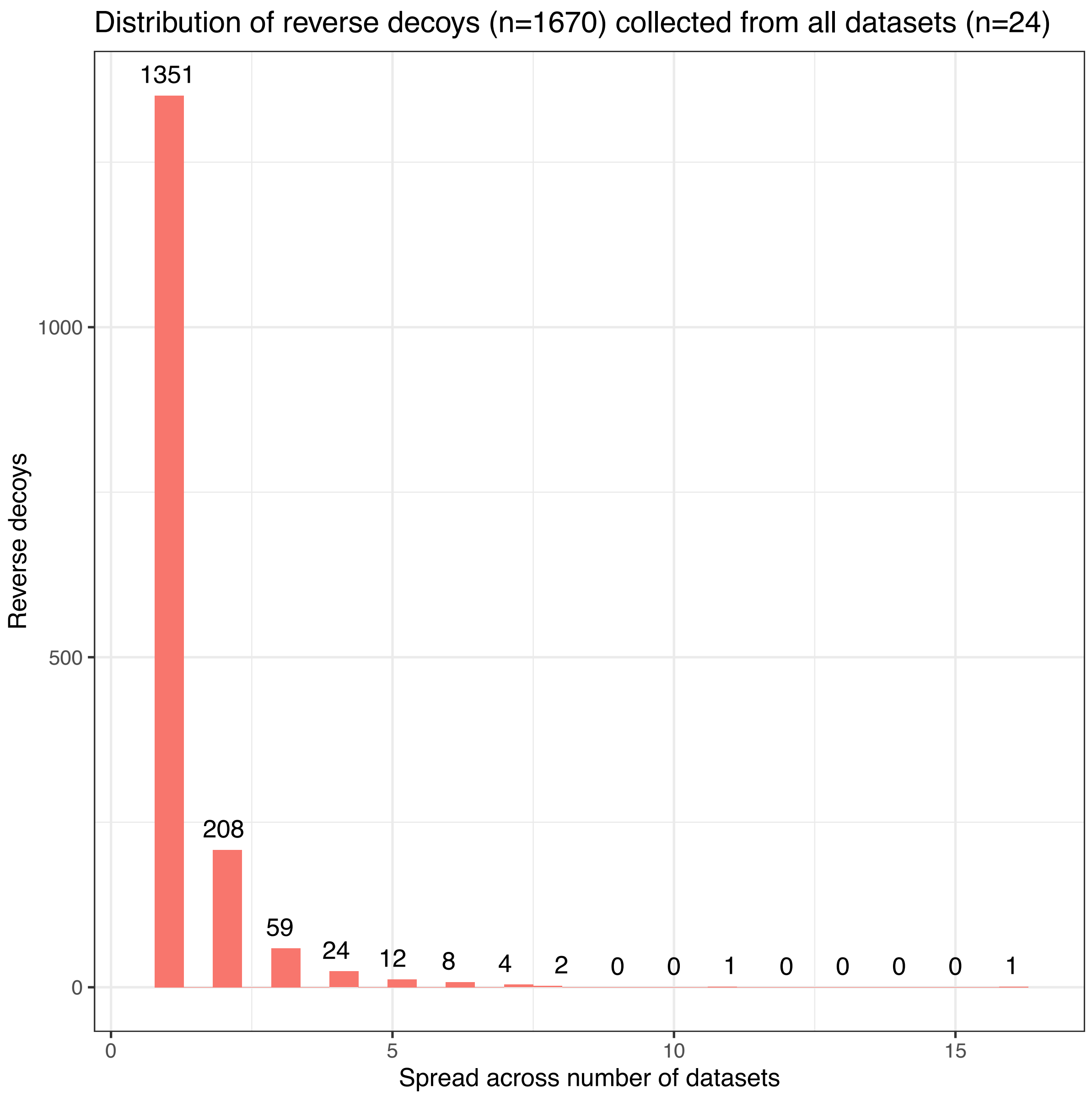

Figure S1. Distribution of common reverse protein decoy hits across the number of datasets.

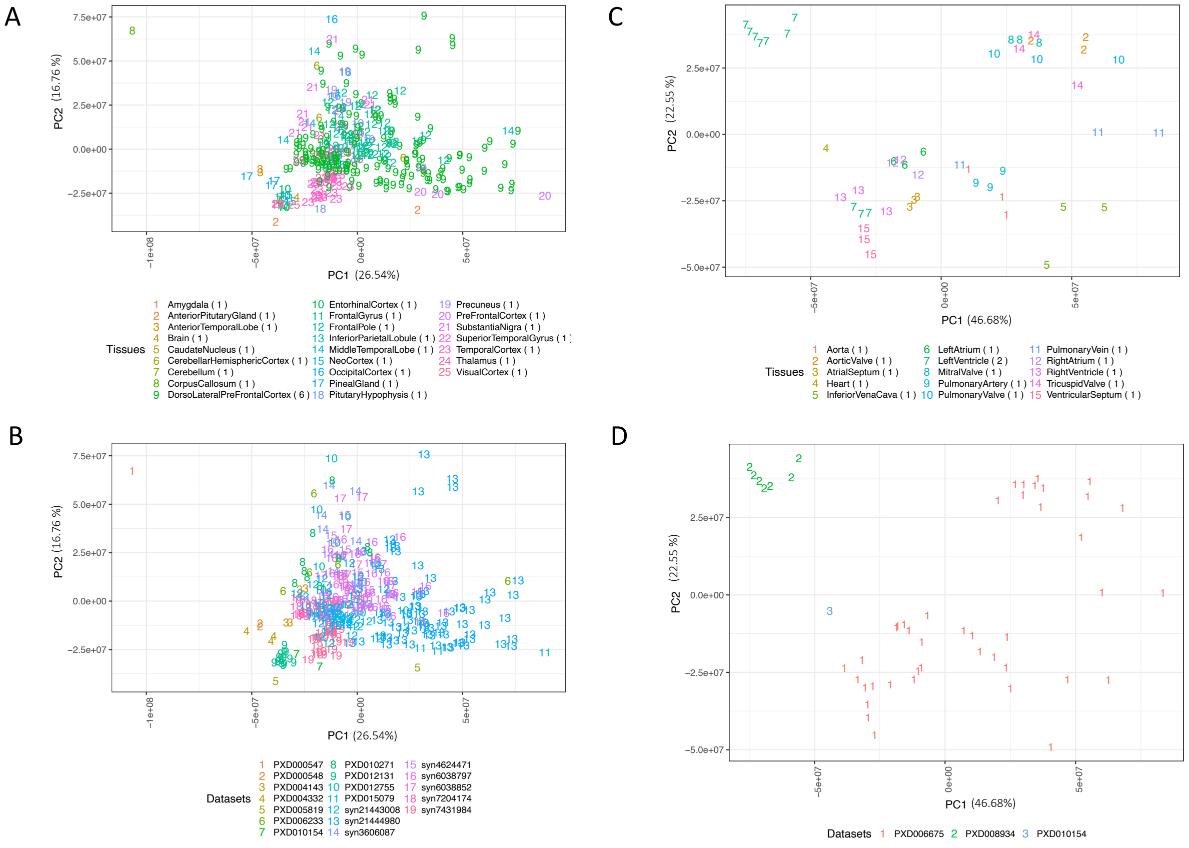

Figure S2. PCA of FOT normalised iBAQ protein abundances (samples without bin transformation) of (A) brain samples coloured by tissues and (B) and datasets (C) and heart samples coloured by tissues and (D) datasets.

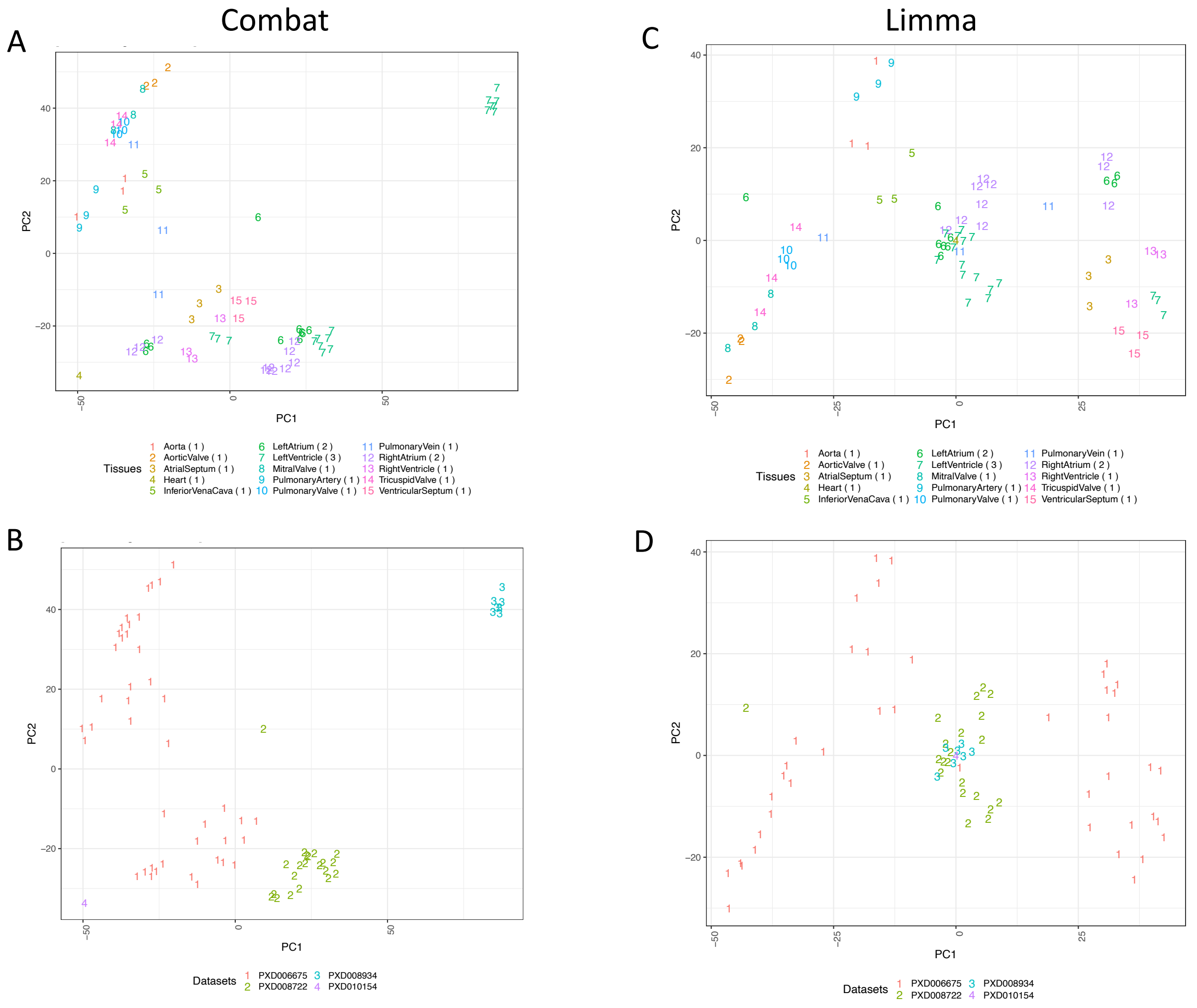

Figure S3. PCA of the heart samples using Combat normalisation where samples are coloured by (A) tissues and (B) datasets. Similarly PCA of heart samples using Limma normalisation where samples are coloured by (C) tissues and (D) datasets.

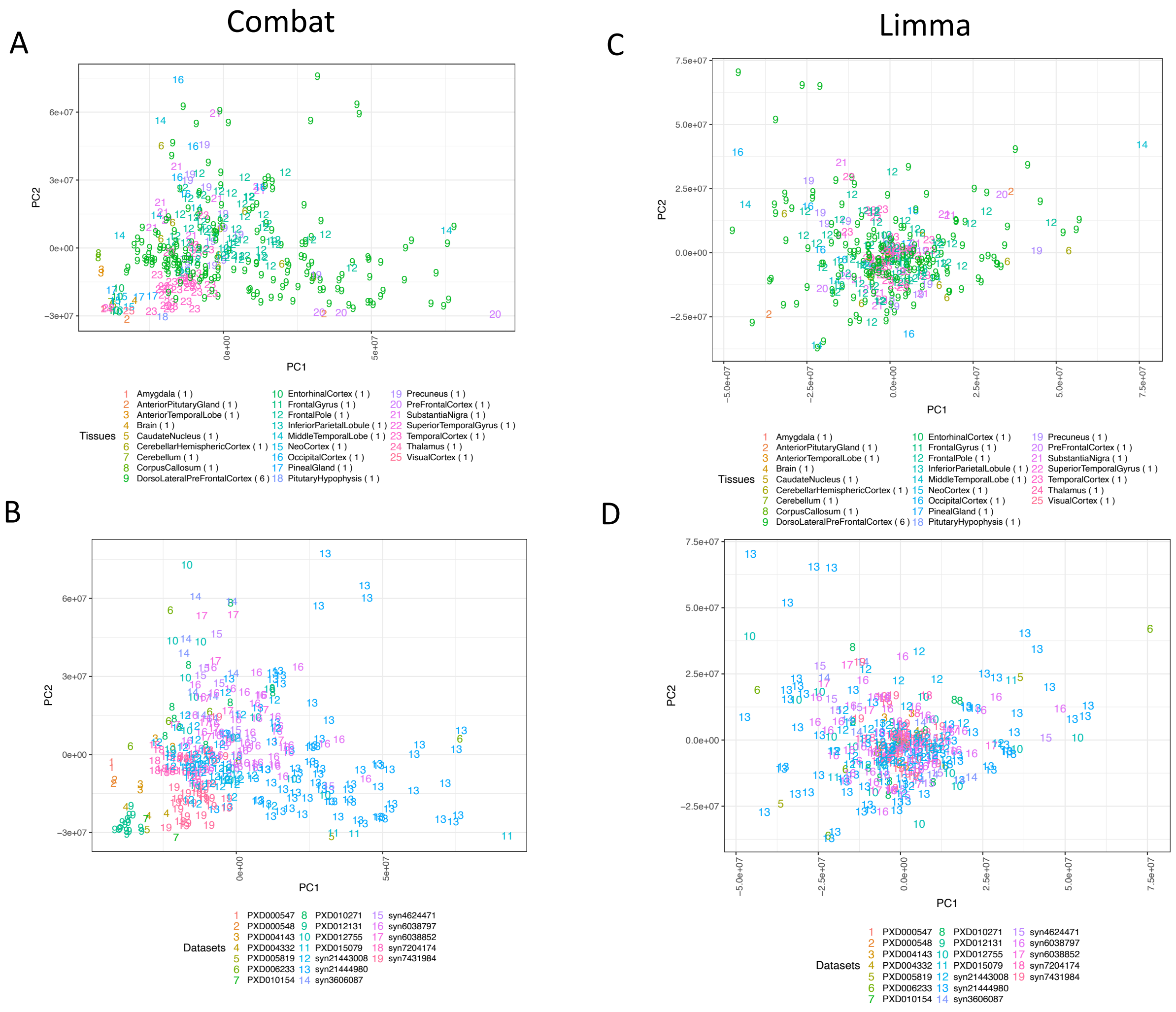

Figure S4. PCA of brain samples using Combat normalisation where samples are coloured by (A) tissues and (B) datasets Similarly PCA of brain samples using Limma normalisation where samples are coloured by (C) tissues and (D) datasets.

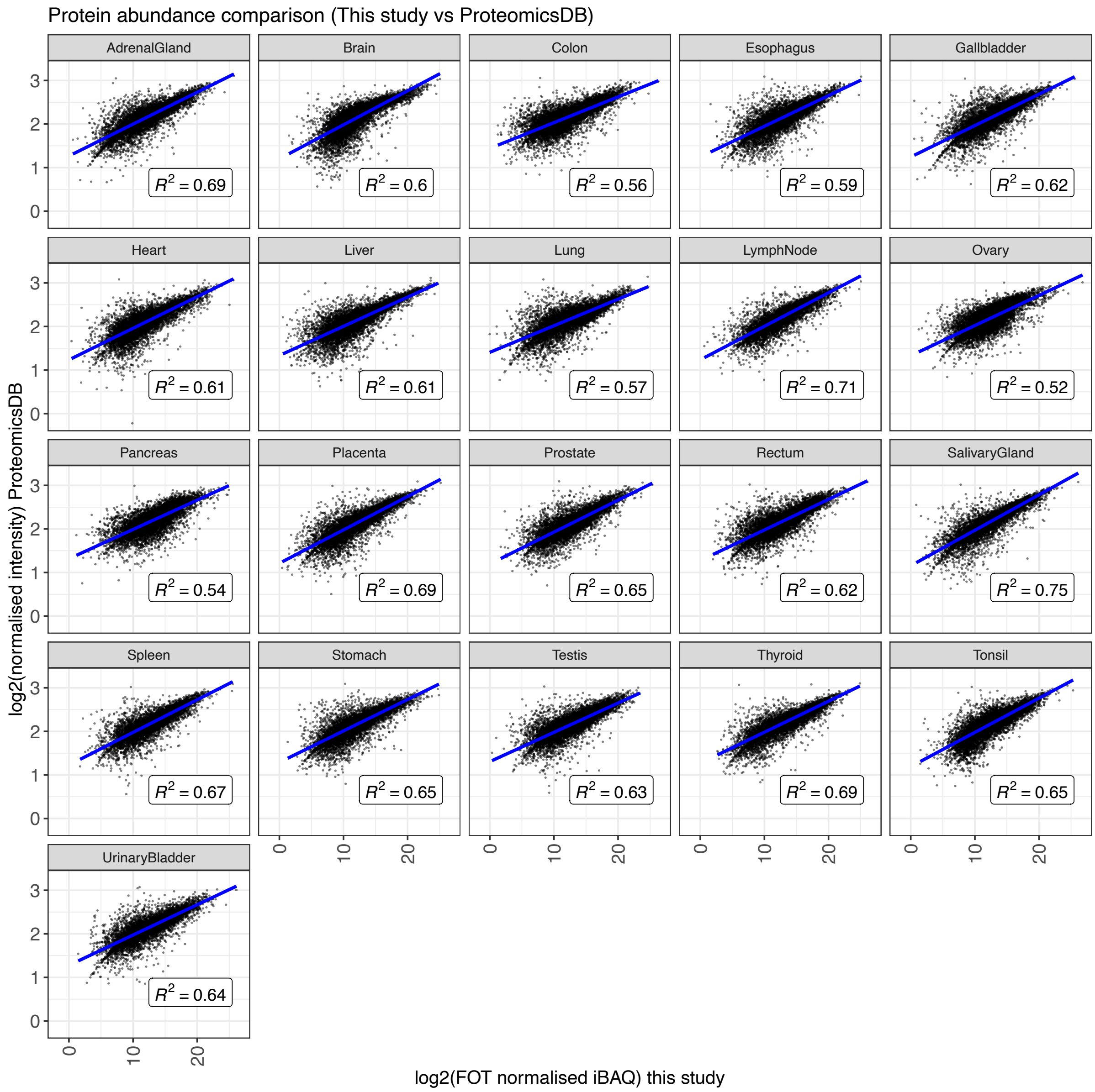

Figure S5. Correlation of protein abundances across various organs compared between ProteomicsDB and this study.

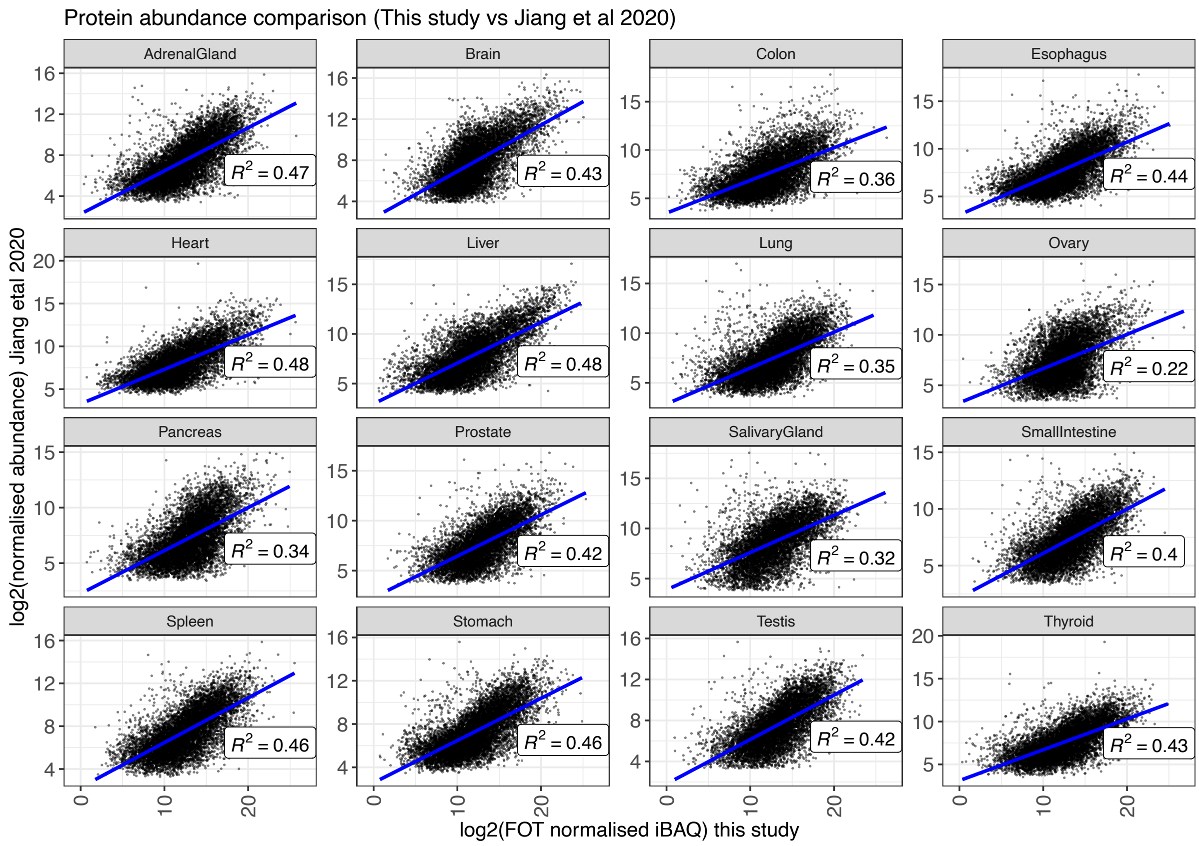

Figure S6. Comparison of protein expression between Jiang et al. (2020) (using TMT labelling method) and data presented in this study (label-free method).

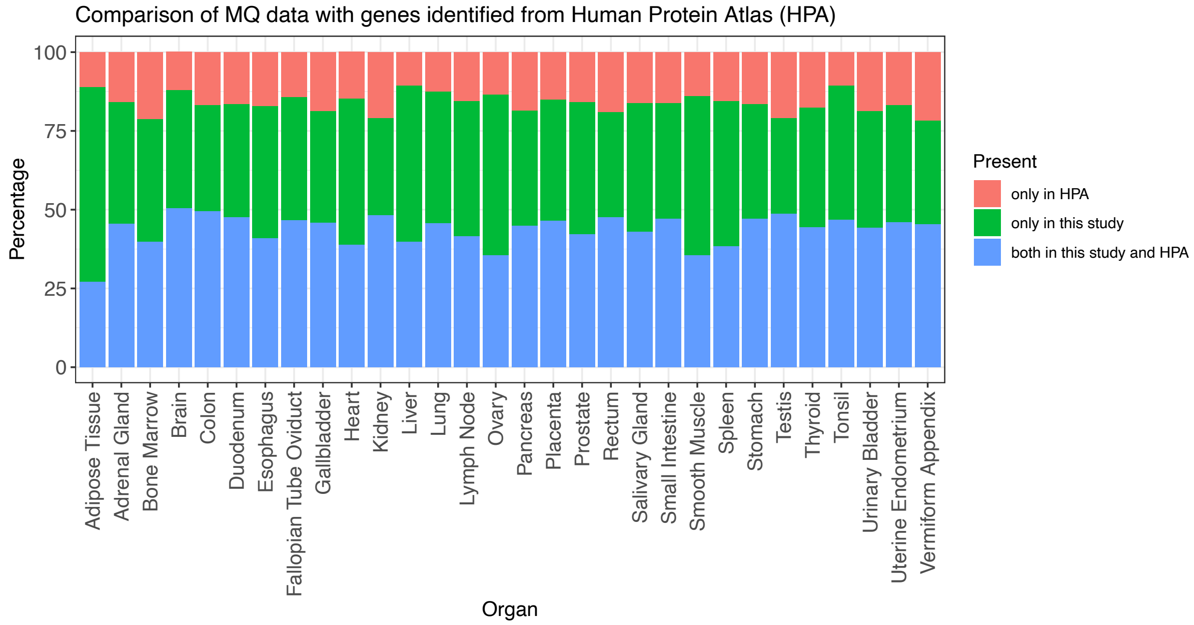

Figure S7. Protein detection comparison between Human Protein Atlas and the results included in this manuscript.

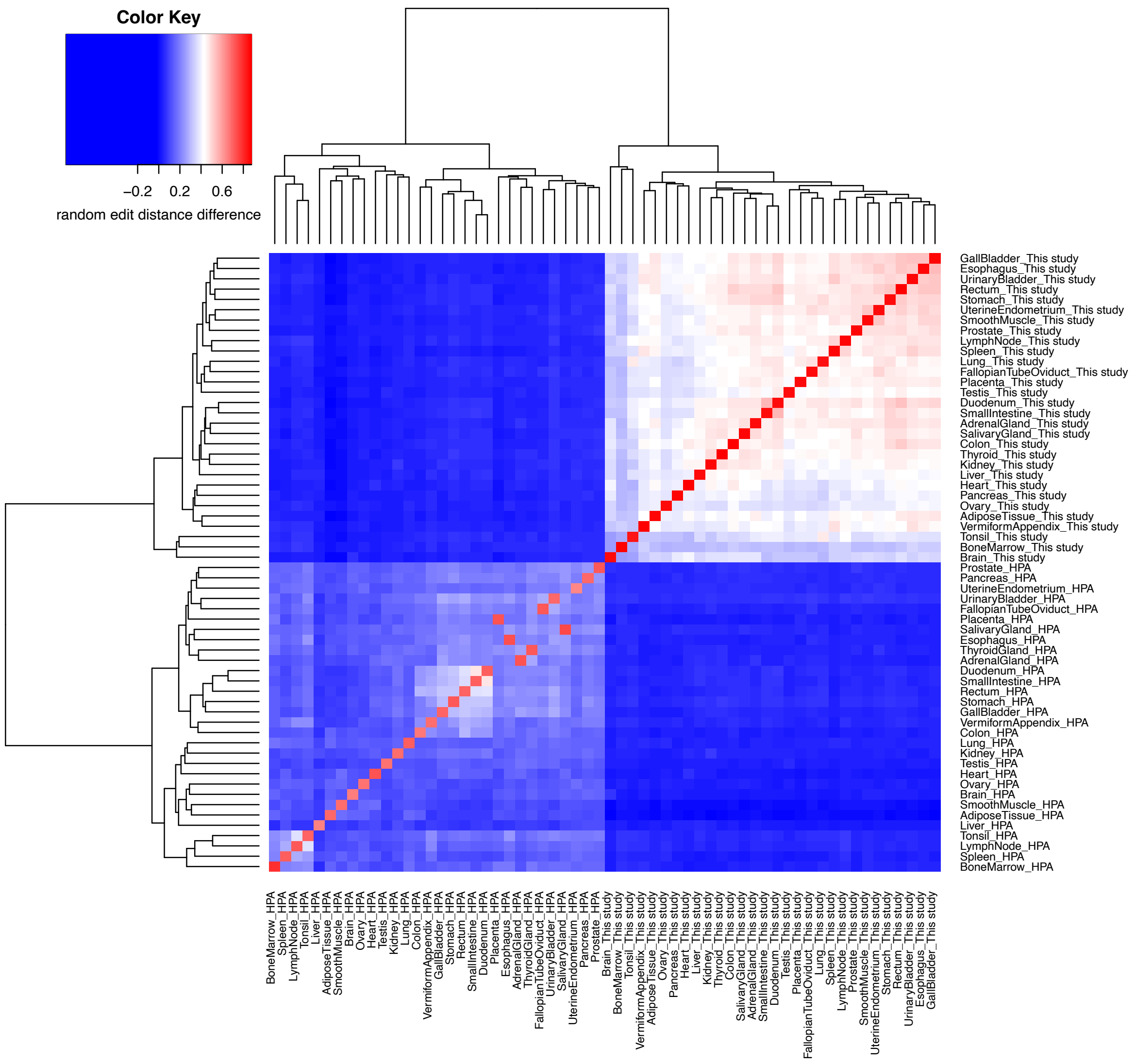

Figure S8. The randomised edit distance difference matrix showing the differences in the edit distances calculated for various organs between this study and the data from Human Protein Atlas.

Browse proteomics experiments in Expression Atlas

You can browse through the experiments in Expression Atlas in the **Browse experiments** tab, which shows you a table listing all the experiments currently available in Expression Atlas. You can filter and/or re-order the table content using the categories and search boxes in the header line.

Proteomics experiments can be listed by typing the keyword proteomics in the **search all columns** field.

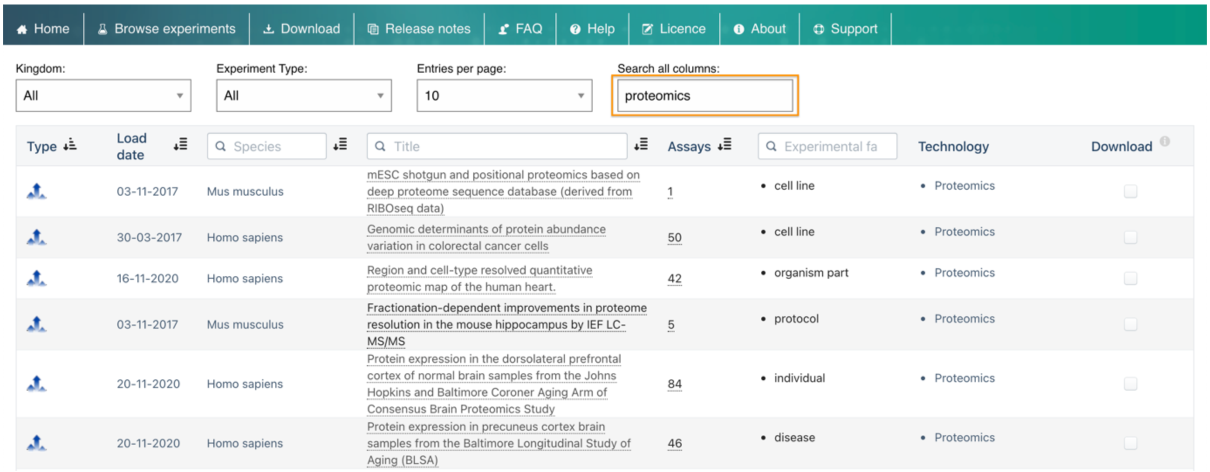

Figure S9. Proteomics experiments home page in Expression Atlas.

Finding an individual dataset

To find an individual experiment, change the experiment accession (example E-MTAB or E-PROT) in the URL www.ebi.ac.uk/gxa/experiments/E-PROT-XX/Results. Replace E-PROT-XX with the proteomics accession of choice (for example E-PROT-52) in the URL.

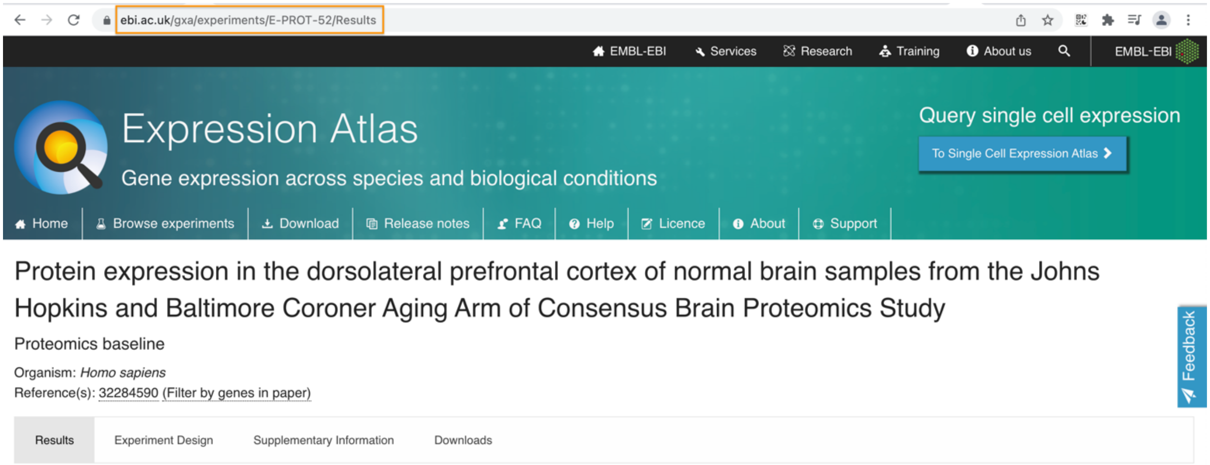

Figure S10. Result page for an example proteomics data. Reprinted (Adapted or Reprinted in part) with permission from [1]. Copyright 2022 EMBL-EBI.

Baseline proteomics experiment page

Each baseline experiment in Expression Atlas has its own Experiment page.

In a baseline experiment page, abundance levels are displayed in one heatmap by colour intensity, according to the gradient bar above the heatmap. The gradient shows intensities corresponding to abundance levels for the 50 genes displayed. Mouse over a cell in the heatmap to see abundance values for each gene in each tissue (or other condition).

For proteomics experiments the abundance values are displayed as parts per billion (ppb). The default minimum expression value is 0.

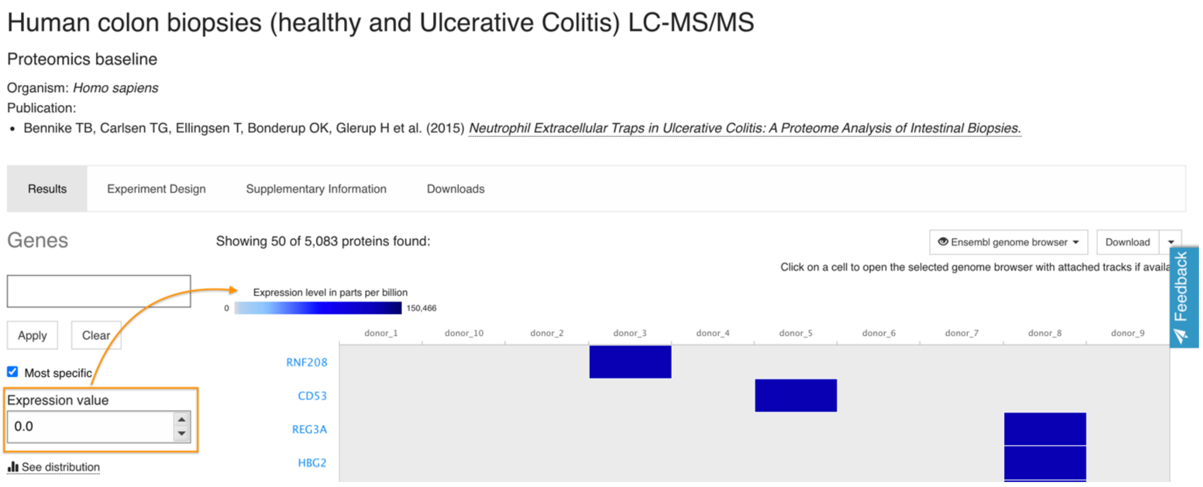

Figure S11. Display of protein abundances across samples in Expression Atlas.

Most specific search

By default, the 50 most specifically expressed genes (rows) across all conditions (columns) studied are displayed. Unclick the **Most specific** option to show genes with highest expression first.

Protein to Gene mapping and quantification

For Mass Spectrometry baseline proteomics experiments the protein abundances are quantified in units of parts per billion (ppb). The abundances of proteins are displayed in terms of their parent gene identifiers. Expression Atlas uses a Gene ID reference frame, therefore to integrate proteomics results the UniProt protein accessions were mapped to Ensembl Gene identifiers using the bioconductor

package 'mygene'. Further details are described in the 'Analysis Method' section of the Supplementary Information to each experiment.

Other information in the baseline experiment page

The **Experiment Design** tab shows mass spectrometry runs along with their corresponding biological sample characteristics and experimental variables values.

The **Supplementary Information** tab for Mass Spectrometry proteomics experiments the analysis methods include data processing protocols for raw Mass Spectrometry data and for post-processing the results, which also includes mapping UniProt protein accessions to Ensembl Gene identifiers.

The **Downloads** tab for Mass Spectrometry based proteomics experiments the files that one can download are: i) raw unprocessed output for baseline Data Dependent Analysis (DDA) experiments, ii) post-processed expression values, iii) quality assessment summary of the experimental runs, iv) input parameters to process raw data files for DDA experiments and v) the experimental design template of all samples.

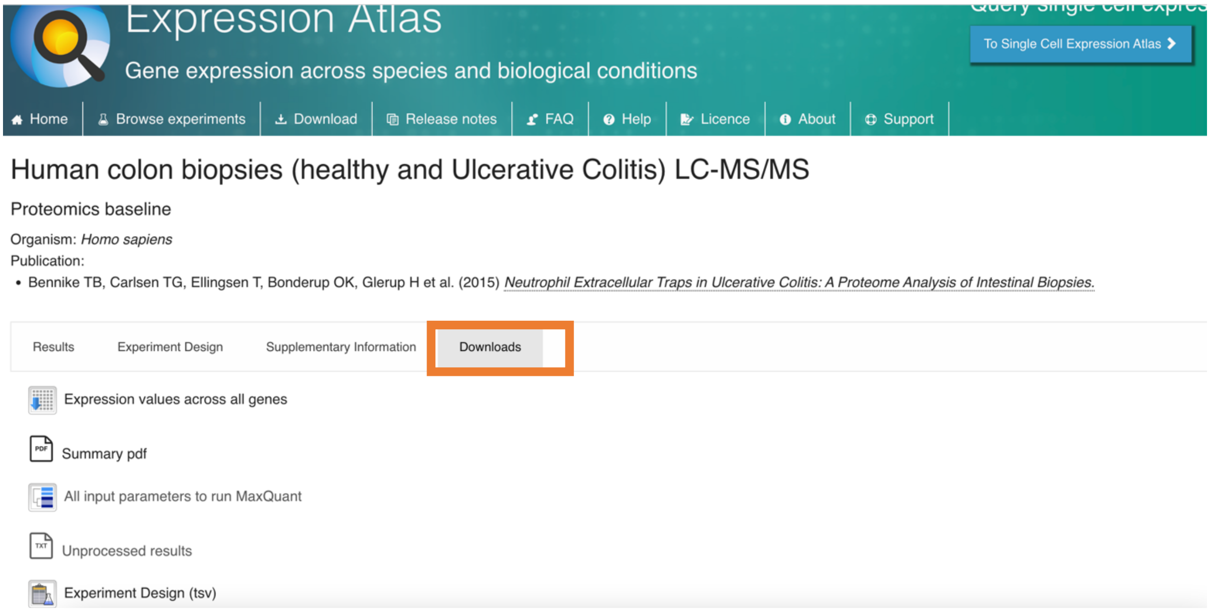

Figure S12. Expression Atlas ‘Downloads’ tab. Reprinted (Adapted or Reprinted in part) with permission from [1]. Copyright 2022 EMBL-EBI.

Supplementary Tables

Supplementary Table 1: Protein groups from all datasets that are mapped to more than one Ensembl Gene ID.

Supplementary Table 2: Median protein abundances (in ppb) for each protein group across various tissue samples in each organ.

Supplementary Table 3: Median binned protein abundances across various organs.

Supplementary Table 4: Table showing the comparison of protein and peptide identification numbers across various datasets with the reported ones in their respective original publications.

Supplementary Table 5: 'Organ-enriched' and 'Group-enriched' elevated proteomes in various organs.

Supplementary Table 6: Gene Ontology enrichment analysis of 'organ-enriched' and 'group-enriched' proteins.

Supplementary Table 7: Reactome pathway-enrichment analysis of "organ-enriched" and "group-enriched" proteins.

References

(1) Papatheodorou, I.; Moreno, P.; Manning, J.; Fuentes, A. M.; George, N.; Fexova, S.; Fonseca, N. A.; Fullgrabe, A.; Green, M.; Huang, N.; et al. Expression Atlas update: from tissues to single cells. *Nucleic Acids Res* **2020**, *48* (D1), D77-D83. DOI: 10.1093/nar/gkz947.
